# The dopamine signal in decision-making tasks with stimulus and timing uncertainty

**DOI:** 10.1186/1471-2202-15-S1-P71

**Published:** 2014-07-21

**Authors:** Stefania Sarno, Víctor de Lafuente, Ranulfo Romo, Néstor Parga

**Affiliations:** 1 Departamento de Física Teórica, Universidad Autónoma de Madrid, Cantoblanco 28049, Madrid, Spain; 2 Instituto de Neurobiología, Universidad Nacional Autónoma de México, Querétaro 76230, México; 3 El Colegio Nacional, 06020 México DF, México; 4 Instituto de Fisiología Celular, Universidad Nacional Autónoma de México, 04510 México DF, México

## 

It was long suggested that the phasic activity of midbrain dopamine (DA) neurons codes the subject’s error in the prediction of reward [1]. Most of the experimental work in this field was done in the context of classical and instrumental conditioning. Recently, there has been an increasing interest to study the activity of DA neurons while the subject performs a decision-making task with the goal of obtaining reward at the end of the trial [2,3]. In an experiment in which monkeys have to decide about the presence or absence of a somatosensory stimulus, recordings of DA neurons have shown that the activity of these cells is modulated according to the trial type. The averaged activity in hit, miss, false alarm or correct rejection trials each presents a distinct temporal profile [3]. In particular, the neurons’ response to the *go cue* is correlated with the subject’s uncertainty about his choice.

The signal of dopamine neurons in classical and instrumental conditioning has been explained in terms of the temporal-difference (TD) algorithm. However it is not clear whether and, if so, how reinforcement learning can account for the dopamine signals in complex decision-making tasks with noisy sensory information and temporal uncertainty of the relevant task events, as is the case in the detection task mentioned above [4]. We have developed an actor-critic model which deals with both these aspects of the problem. While an internal temporal representation keeps track of past relevant events, partial observability is accounted for by means of a Bayesian approach.

The dopamine phasic activity predicted by the model matches the experimental data and the prediction of the psychometric curve is consistent with the animal performance. Furthermore, the model provides an interpretation of the condition-dependent dopamine response to the go cue instruction in terms of reward prediction error. Using Bayesian inference the model constructs an internal belief about the presence of the somatosensory stimulus. This belief reflects the confidence about the sensory perception and thus the value assigned to this perceptual judgment. The large belief in stimulus-present decisions represents a high degree of confidence in the sensory perception and a great expectation for future rewards. On the contrary, stimulus-absent choices reflect a small belief and consequently a larger uncertainty about the decision and the future reward. This computational description of belief agrees with the previous interpretation of the data [3] and describes well other experimental observations such as the dependence of DA neurons' signals on the stimulus amplitude. The model also predicts a decrease in dopamine activity before the go cue instruction, which is also observed in the data. We explain this decreasing tonic activity as an effect of the timing uncertainty. This is partly due to the task structure and partly generated from the limited temporal resolution of the stimulus representation, which creates subjective variability in the timing of events.
